# Assessment of Needs in Children Suffering From Refractory Non-neurogenic Urinary and Fecal Incontinence and Their Caregivers' Needs and Attitudes Toward Alternative Therapies (SNM, TENS)

**DOI:** 10.3389/fped.2020.00558

**Published:** 2020-09-09

**Authors:** Joana Dos Santos, Edyta Marcon, Martha Pokarowski, Reza Vali, Lucshman Raveendran, Fardod O'Kelly, Afsaneh Amirabadi, Dean Elterman, Richard Foty, Armando Lorenzo, Martin Koyle

**Affiliations:** ^1^Division of Urology, The Hospital for Sick Children, Toronto, ON, Canada; ^2^Translational Research Program, Laboratory Medicine and Pathobiology, University of Toronto, Toronto, ON, Canada; ^3^Donnelly Centre, University of Toronto, Toronto, ON, Canada; ^4^Diagnostic Imaging, The Hospital for Sick Children, Toronto, ON, Canada; ^5^Institute of Health Policy Management and Evaluation, University of Toronto, Toronto, ON, Canada; ^6^Krembil Research Institute, University Health Network, Toronto, ON, Canada

**Keywords:** child health, non-neurogenic refractory incontinence, quality of life, sacral neuromodulation, transcutaneous nerve stimulation

## Abstract

**Background:** Non-neurogenic urinary and fecal incontinence (UI, FI) affects approximately 6% of North American children with 1% of cases becoming refractory (nonresponsive to standard therapies). Incontinence has major potential long-term physiological and psychological implications for patients and their families. While Sacral Neuromodulation (SNM) and Transcutaneous Nerve Stimulation (TENS) are alternative therapies available for the treatment of refractory UI/FI, these are not approved for use in children in Canada. The present study assessed participants' perception of current treatments, incontinence burden, and attitudes toward novel therapies in a single pediatric institution.

**Methods:** Multiple validated questionnaires including Dysfunctional Voiding Scoring System (DVSS), Bristol Stool Chart (BSC), Pediatric Incontinence measurement (PinQ), and Time-Driven Activity Based Costing were used to perform a needs assessment for patients with non-neurogenic refractory incontinence, and to determine patients' and caregivers' attitudes toward alternative therapies.

**Results:** 75% of patients and 89% of caregivers reported a moderate to severe impact of incontinence on QoL with diminished social interactions among the primary concerns. Caregivers were frustrated with current treatments and were open to trying alternative therapies (SNM and TENS), which, at least in the case of SNM, seems to be less expensive, possibly less burdensome and more effective than current surgical options.

**Conclusion:** Pediatric refractory UI/FI has a large impact on patients' and caregivers' QoL and alternative therapies with the potential to improve QoL of patients and caregivers should be further investigated as a substitute for surgery.

## Introduction

Non-neurogenic urinary and/or fecal incontinence (UI/FI) refers to the involuntary leakage of urine or stool in the absence of central or peripheral neurological causes. Approximately 10% of adults and 6% of children in both Canada and the US (1–3) suffer from these conditions, however the actual prevalence is likely underreported due to the social stigma associated with incontinence. The most common cause of non-neurogenic incontinence in children is Bladder and Bowel Dysfunction (BBD), which can be responsible for up to 40% of pediatric urology clinical visits (4). BBD describes a multitude of lower urinary tract symptoms (LUTS), often accompanied by constipation and/or encopresis. BBD is also commonly associated with vesicoureteral reflux (VUR) and recurrent (febrile) urinary tract infections (UTIs), which can ultimately impact renal function (5). Incontinence also affects children's psychosocial well-being including anxiety, self-esteem, shame, isolation, poor school performance, and other behavioral changes (4).

Standard therapy for both children and adults with non-neurogenic UI/FI in Canada includes behavioral and dietary changes, biofeedback, and pharmacological treatments (6). Medications, in particular antimuscarinics, may elicit adverse effects (dry eyes, dry mouth, constipation, GI upset, heat intolerance) but can provide effective treatment outcomes in most patients. Despite the success of these therapies, 1% of patients will become refractory and will not respond to any treatment (7, 8). Currently, the only options for refractory cases have been surgical. These include botulinum toxin injections for urinary incontinence and transanal irrigation and anterograde continence enema for fecal incontinence. However, these surgical alternatives can be burdensome for the patient, are not always successful and carry their own risks of morbidity (9–11).

Incontinence-afflicted adults who do not respond to first-line therapies are eligible for sacral neuromodulation (SNM) or transcutaneous nerve stimulation (TENS). SNM and TENS are minimally invasive and reversible treatments that rely on delivering electrical stimulation to the S3 foramen or saphenous nerve, respectively. In the US, SNM is considered standard of care for adults suffering from non-neurogenic refractory incontinence while in Canada, SNM is performed on case-by-case basis. SNM and TENS are not approved by the FDA or Health Canada for use in children even though international studies have shown that SNM therapy can positively affect symptoms associated with constipation and urinary dysfunction, decreases reliance on pharmacological interventions and improves QoL in children (12–23). TENS is associated with immediate and short-term improvement in children suffering from nocturnal enuresis, although there is a paucity of data in the contemporary literature (21, 24).

It is essential to investigate these therapies as a potential treatment for non-neurogenic refractory incontinence in children as an alternative to current surgical options. While SNM is considered a minimally invasive procedure, it requires an operating room and an implantation of a neurostimulator that sends electrical signals to the sacral nerves, as such, it is important to understand patient willingness to undergo these procedures. This study aims to determine the impact of non-neurogenic refractory UI/FI on children and their caregivers, assess patient's and caregiver's attitudes toward the potential use of SNM/TENS therapies in Canada and to provide a preliminary cost analysis of SNM therapy vs. standard surgical options within the Canadian context.

## Methods

### Study Design, Population, and Recruitment

This was a single-center, cross-sectional study of patients with non-neurogenic refractory UI/FI followed at the urology outpatient clinic of a quaternary care pediatric hospital in Ontario, Canada. Following institutional Research and Ethics Board approval (REB# 1000058568), we prospectively recruited patients who presented with non-neurogenic refractory incontinence and their caregivers from January to August, 2018. All patients were evaluated with videourodynamics (refractory UI only) and spine MRI (refractory UI and/or FI). Patients with abnormal findings on MRI were excluded from this study. We defined refractory incontinence as persistent UI/FI after 6 months of conservative management (bladder retraining and constipation treatment ± biofeedback and pelvic floor physiotherapy), followed by lack of response to adequate medical therapy (maximized antimuscarinics ± beta-3 adrenoceptor agonists; or alpha-blockers for dysfunctional voiding with high post void residuals) for at least 3 months in patients aged 5–17 years. We included patients who regularly attended our institution for treatment of BBD, LUTS, voiding dysfunction, urinary retention, constipation, and UTI; spoke English and; could provide consent. We excluded patients with spinal cord injury, developmental delays, behavioral/psychiatric disorders, immunodeficiency, bleeding disorders, and inflammatory bowel disease; did not speak English; or were unable to provide consent. Families were recruited during scheduled visits. After obtaining consent, patients completed the Dysfunctional Voiding Scoring System (DVSS), Bristol Stool Chart (BSC), the Pediatric Incontinence measurement (PinQ) tool for children (or the parental proxy), and a needs assessment questionnaire with open-ended questions (Supplementary Appendices A, B) (12, 13, 25–27).

### Measurement Tools

DVSS is a validated 10-parameter instrument used to measure pediatric non-neurogenic dysfunctional voiding. DVSS scores of ≥6 for females and ≥9 for males are indicative of incontinence (26). The BSC detects the presence of functional defecation disorders and has been validated for use in children (27). The PinQ is a cross-culturally validated test with proven test-retest reliability and good parental proxy, developed for non-neurogenic enuretic children to evaluate social, self-esteem, family function and body image domains of QoL (12, 13, 25). A mild, moderate or severe impact of incontinence on QoL is defined by a PinQ score of <20, 20–50, or >50, respectively (28). Descriptive statistics were used to evaluate demographic data and questionnaire scores. The Mann-Whitney/Wilcox test was used to assess differences between patient and caregiver PinQ scores.

### Qualitative Analysis

Patient and caregiver responses to open-ended questions were de-identified and transcribed from written to electronic text. Codes were generated manually by two independent investigators and inconsistencies were discussed and resolved. Responses were analyzed using NVivo software (NVivo 12, QSR International, Cambridge, MA).

### Time-Driven Activity-Based Cost Analysis

TDABC calculates costs by combining the capacity cost rates (CCRs) of key direct and indirect resources as well as time estimates from electronic medical records (EMR) for processes involved in patient care (29). Direct resource costs include the cost of providers and material resources. Indirect costs include the cost of using hospital spaces which includes general administrative and overhead support activities. To calculate capacity cost rates, the total costs of staff, supplies, and clinical working spaces were captured using budgets and salary information from the 2018–2019 fiscal year divided by the total available hours of those resources for patient care. Time estimates from EMR data were then applied to generate costs for each step of the procedure. TDABC model includes cost of OR and PACU space, materials (device, drugs, equipment, etc.), and the cost of non-physician staff (OR nurses, etc.). Physician costs are not included in this model. Similarly, follow up costs are also not included. For SNM the costs for one stage and two stage procedures are provided. All costs are provided in Canadian dollars.

## Results

### Study Participants

Of the 30 patient families approached, 29 agreed to participate in this study (96.7%). The median age of patients was 9.6 (range 5–15) and 79% were female. Of the 21 patients with siblings, 32% of those siblings also had issues with incontinence (Table 1). The median DVSS of patients as reported by their caregivers was 12 (range 2–23; Table 2) and 61% of children attained a DVSS score >11 indicating at least moderate incontinence. There was no statistically significant difference in DVSS with sex, patient age, parental marital status, or household income.

**Table 1:** Characteristics of study population.

Patients (*n* = 29)	Gender	Male	6 (21%)
		Female	23 (79%)
	Mean Age	9.6 years (5–15 years)
Parents (*n* = 29)	Marital Status	Single	0 (0%)
		Married/Common Law	24 (83%)
		Divorced	5 (17%)
	Employment Status	Employed	22 (76%)
		Not employed	7 (24%)
	Parents with >1 child		21 (72%)
	Parents with >1 child with UI		7 (32%)

**Table 2:** Participants' DVSS and QOL scores.

	**DVSS**	**QOL**
Parents	NA	36.50 (5–56)
Patients	12 (2–23)	32 (2–50)

*DVSS scores of the children were based on parental responses*.

Twenty-eight patients and their caregivers (96.5%) completed the PinQ. Patient PinQ results showed no significant difference in QoL with sex (Table 2). Parental PinQ results showed no significant differences with patient age, sex, or parental marital status. Overall, 71% of patients and 89% of parents reported moderate to severe impact of incontinence on QoL (PinQ ≥20). Patient and parental PinQ scores were strongly correlated (*p* < 0.0001, rho = 0.758), but no significant correlations were observed between PinQ score and patient's age or caregiver's marital status.

### Needs Assessment

**Figure 1:**
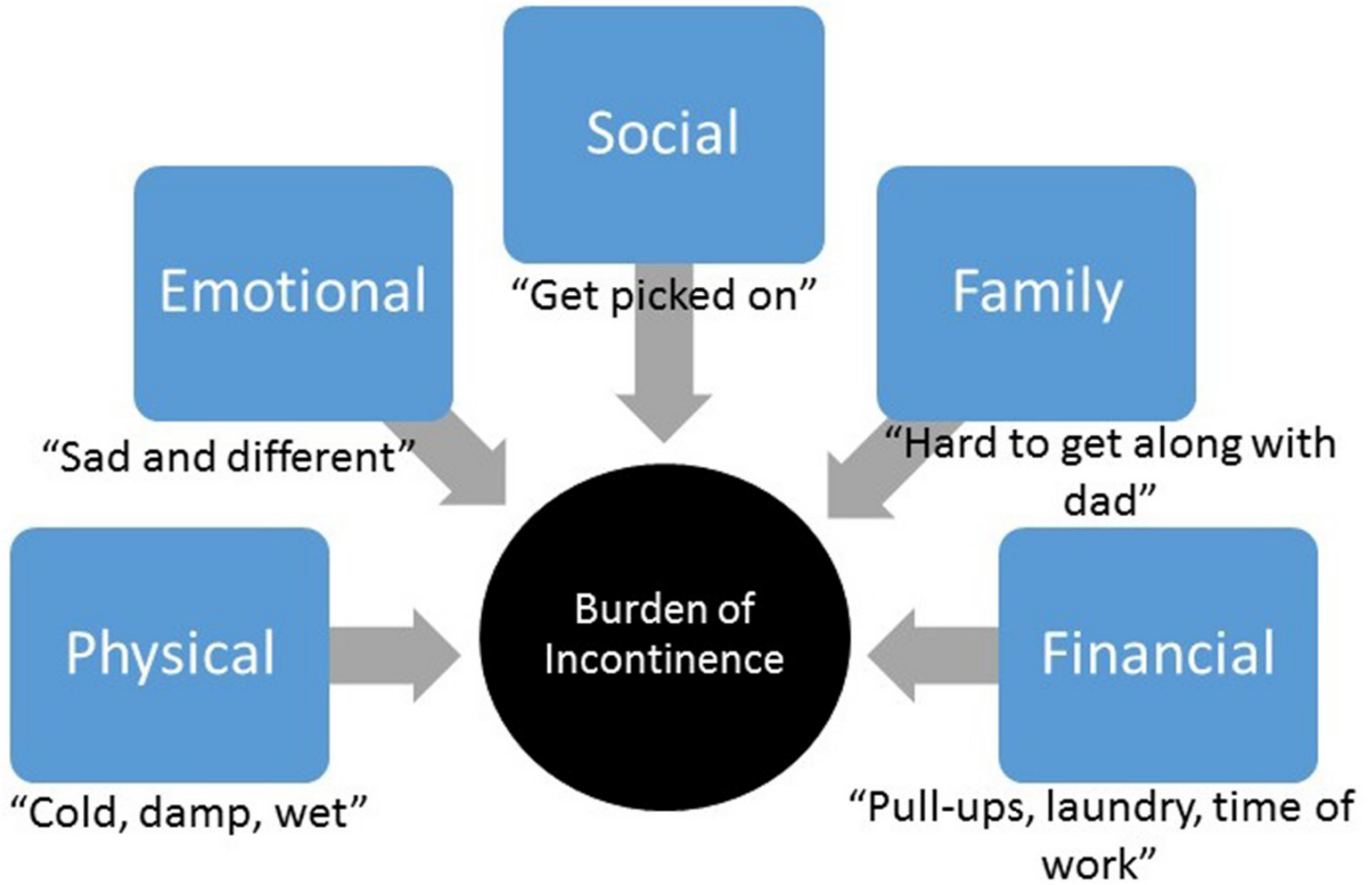
Incontinence burden. Major themes identified within patients' and parents' responses related to incontinence burden.

Based on patients' responses, incontinence burden was grouped into five major themes: physical, emotional, social, family, and financial burden (Figure 1).

#### Physical Burden

**Figure 2:**
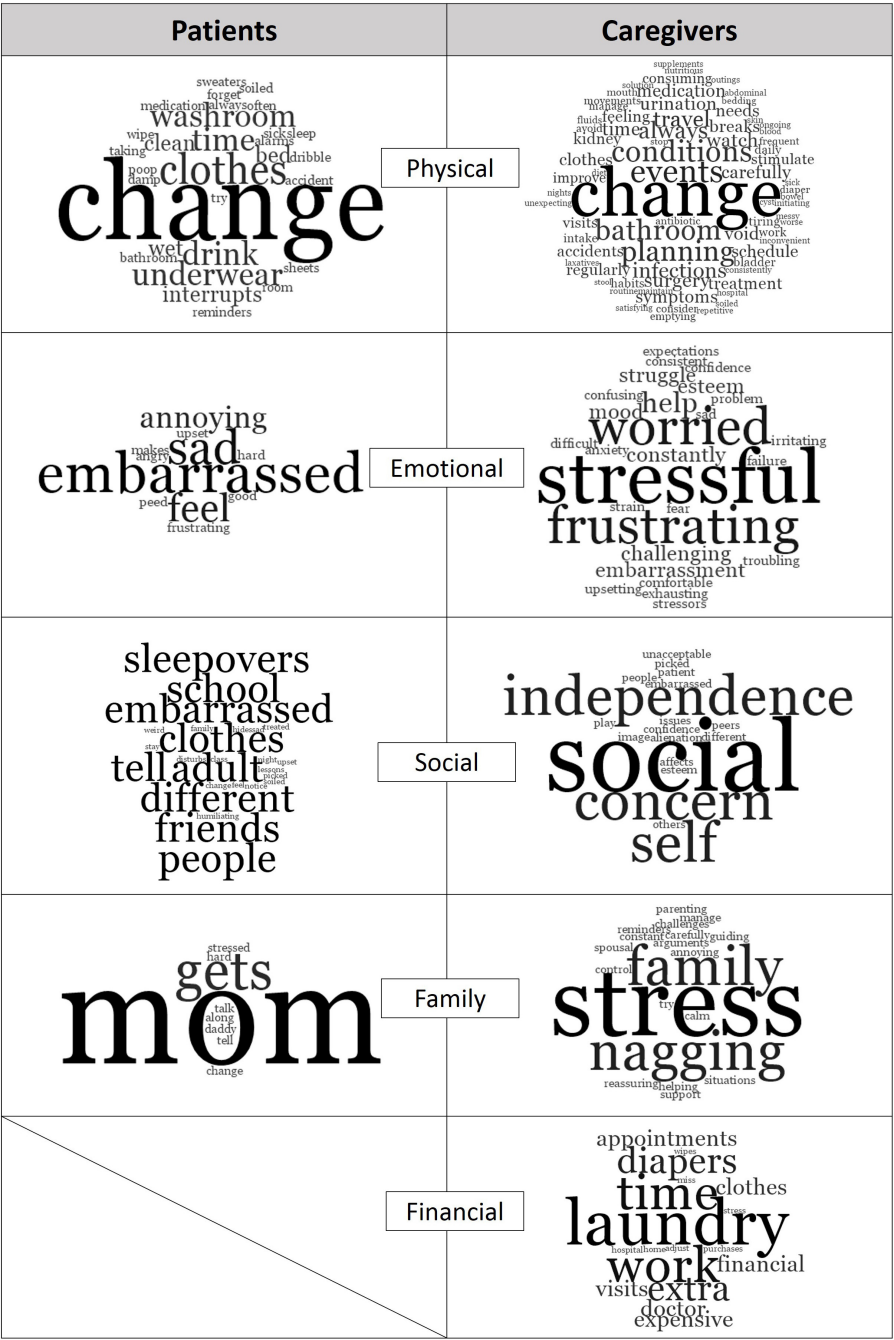
Study themes and most common words mentioned by patients and caregivers. Generated with NVIVO.

“Change” was the predominant word in this theme (Figure 2). Patients reported the need for frequent change of clothing, restricting fluid intake, going to the washroom before bed and setting alarms. Caregivers reported frequent clothing changes and stressed the need to always be prepared which resulted in feelings of tiredness: “This can be tiring, (and) time consuming to always have to change him and bring clothes and pull-ups everywhere.” Caregivers often monitored the frequency of their children's washroom visits and encouraged proper diet and voiding habits, which could be “exhausting,” as one caregiver described it. Caregivers also worried about the long-term consequences of incontinence such as infections, impact on future kidney function, medication use, treatments, and the potential need for surgery.

#### Emotional Burden

Incontinence took an emotional toll on both patients and caregivers. Caring for a child with refractory UI/FI contributed to lost sense of control, heightened stress and increased frustration. Patients expressed feeling “different,” “embarrassed,” “sad,” and “angry” (Figure 2). Patients also reported feeling bad for their caregivers: “I was sad when I peed (the) bed and daddy had to change.” Caregivers reported wanting to control situations and being frustrated and anxious when it was impossible. Caregivers also worried about their children's emotional well-being and did not want to see them feeling embarrassed, ashamed or guilty. When discussing their daughter, one parent reported the “stress of feeling like she (the daughter) had done something wrong.”

#### Social Burden

Both patients and caregivers expressed that refractory UI/FI contributed to a loss of social interaction: “It's hard missing out on things with my friends,” “I can't go to sleepovers,” “(I) Don't have many friends because of (incontinence),” and “(I) get picked on” (Figure 2). Social alienation, lowered self-esteem, reduced independence, and future implications were also the major concerns of caregivers. Caregivers also worried about their children's self-esteem, and how “(incontinence) affects (their child's) confidence outside of home,” “I am worried he'll get picked on.”

#### Family Burden

The most prevalent word among patients with respect to incontinence management was “mom” (Figure 2): “My mommy wakes me,” “My mom changes me and cleans up,” “talk with mommy,” “find solutions with mommy for school and friends,” and “tell mommy and she helps.” Patients also reported that incontinence is stressful on their caregivers and on family life in general: “mom gets stressed,” incontinence “makes it hard to get along with daddy sometimes.” Stress was the predominant descriptor used when caregivers talked about their children's incontinence in relation to family life. Caregivers constantly monitored and reminded their children to visit the washroom frequently, ensure soiled clothes are changed, and encourage appropriate exercises: “I feel for my daughter. I don't like to see her embarrassed and don't enjoy nagging (her) to drink more or use (the) washroom.” Still, another caregiver summed it up simply as, “you do what has to be done for your child.”

#### Financial Burden

Approximately 61% of parents reported that their child's UI/FI is a significant financial burden, primarily due the cost of incontinence-related items and missed work. Laundry was the most common word in this theme (Figure 2). One caregiver expressed choosing buying diapers over groceries when money was tight. Caregivers reported taking time off work for hospital visits or to stay home when necessary. One caregiver reported “(I) have lost weeks worth of pay to be in the hospital to talk with doctors.” Another reported “(I) lose sleep and miss work.”

### Current and Alternative Treatments

The majority (72%) of caregivers reported limited, no improvement or a “cycle of improvement and setbacks” with current treatments. Caregivers were hopeful new treatments may be helpful in reducing their child's “on-going symptoms,” or limit their child's antibiotic intake. Others were hopeful new treatments may improve their child's QoL, self-esteem and make them “feel normal.” One caregiver stated that a new treatment is “worth trying even if it doesn't cure.” Over half (54%) of caregivers were open to trying TENS therapy. Of those, 42% were also open to trying SNM therapy. Approximately 21% of caregivers were unwilling to try either therapy while 25% indicated they were unsure or required additional information.

### Cost Analysis

A preliminary cost analysis was performed to compare the surgical costs of SNM therapy vs. common established treatment modalities for children with refractory non-neurogenic urinary and fecal incontinence which include but are not limited to botulinum toxin injections for urinary incontinence, open/ laparoscopic caecostomy for antegrade enemas, and retrograde transanal irrigation systems for fecal incontinence. Surgical costs were calculated using the time-driven activity-based cost analysis (TDABC) methodology (29). Costs are given in Canadian dollars, using Canadian public health system, and based on our institutional experience and available literature. The costs do not include physicians time, follow up appointments, ongoing costs into adulthood (30, 31), nor the time required daily for personal care (>1 h/day). The costs indicated here include the initial surgery/set up and supplies for 3–5 years. The preliminary longitudinal cost assessment indicates that SNM therapy is less expensive than current standard surgical options for both UI/FI (Table 3). This is mainly due to the continuous need for re-administration, or significant accumulative daily costs for equipment/materials (catheters/flushes) required with the current options.

**Table 3:** TDABC of costs for management of refractory urinary and fecal incontinence.

**Sacral Neuromodulation (SNM) ($CAD)**		**Standard Treatment Options ($CAD)**		
SNM Lead Test & Implantation One-Stage	$11,930	Urinary Incontinence	Botulinum Toxin Surgery^*^	$9,030–15,050
SNM Lead Test & Implantation Two-Stage	$13,269	Fecal Incontinence	Transanal Irrigation System^+^	$14,198–23,664 (32, 33)
			Malone Anterograde Continence Enema^x^	$6,433–8,547 (34–36)

* *Two injections/year over 3–5 years*.

+ *System use over 3–5 years*.

x *Includes daily flushing over 3–5 years*.

## Discussion

Refractory non-neurogenic incontinence is complex, with short- and long-term consequences that affect patients and their immediate circle of care. Two-thirds of patients indicated that incontinence has a moderate to severe impact on their lives. The remaining one-third expressed minimal impact and consisted of patients aged <12. Younger children are more dependent on their parents and thus the loss of independence, friends, and sleepovers, may play a smaller role. Bedwetting has been shown to lead to behavioral problems and both wetting and bowel accidents carry social stigma that may affect self-esteem and feelings of shame and isolation (2, 4, 28). Bullying, name-calling and having few friends affect not only the child's self-esteem but also his/her social development and academic performance at school. Less is known about the parental burden of a child's refractory UI/FI. Studies that examined the impact of parental well-being in similar populations indicate a general decrease in QoL (5, 6). In this study, pressures on caregivers led to altered family dynamics, marital discourse, and financial burden, consistent with previous publications (28).

As a result, 42 and 54% of caregivers welcome the potential use of novel therapies, SNM and TENS, respectively. Caregivers were more reluctant to try SNM, presumably due to its invasiveness. Several caregivers indicated that they require more information about SNM and TENS, suggesting these therapies are not well described as treatment alternatives. Twenty-one percent of caregivers were unwilling to try either SNM or TENS. This may be due to the length of time children struggled with this condition. While all the children enrolled in the study have previously tried multiple non-invasive therapies, some have suffered for less than a year while others struggled with the condition for much longer. The shorter time since the diagnosis might have led some parents to still hope that the condition will resolve itself and their unwillingness to try invasive therapies. In addition, parents of younger children might have also hoped that the condition will resolve itself without invasive treatments. Due to the small sample size of our study, we were unable to determine correlations between children's age, condition persistency, and willingness to try SNM or TENS. Moreover, while caregivers were asked about their willingness to subject their children to SNM or TENS therapies this question did not relate SNM or TENS therapies as alternatives to surgical options (Botox injection or MACE). It is possible that if provided with a choice, parents will more readily opt for SNM or TENS. Approximately 50% patients were willing to undergo SNM or TENS, indicating that caregivers are willing to try anything to ease suffering for their children.

One potential advantage of the SNM system is that it demonstrates efficacy in treating both fecal and urinary incontinence, as opposed to current standard surgical options which may require a combination of multiple surgical therapies to treat this dual issue. In our institution, botulinum toxin injections are a standard surgical option used to alleviate symptoms of urinary incontinence (associated with refractory overactive bladder, small bladder capacity or low compliance) while transanal irrigation and MACE are surgical options used to alleviate symptoms of fecal incontinence. SNM has demonstrated higher success rates compared to botulinum toxin surgery in improving symptoms of urinary incontinence but also leads to the decrease and finally elimination of antegrade continence enema use in children with severe constipation (19, 23, 37, 38). This point must be considered in the context of conventional treatment failure which is associated with high costs of health care resource use (ER visits, clinic visits), and community costs including the cost of continence supplies which could be in excess of CAN$2000/year for some families (3, 34, 39, 40). Furthermore, once permanently implanted, SNM does not require further repetitive treatments (as opposed to Botulinum toxin injections) or multiple episodes of general anesthesia, and does not have the same revision risks and side effect profile seen with cutaneous abdominal stomas (41). Morever, botulinum neurotoxin injections can lead to antibody production in patients and subsequent therapy failure (42). This, of course is not a factor in SNM therapy. This preliminary cost description can be used in future cost-effectiveness work that accounts for health outcomes, community costs, and transition of health states, which is needed to definitively establish the cost-effectiveness of the SNM implantation in this population.

This study was conducted in a major Canadian pediatric center where SNM has been demonstrated to have fewer outright costs than current standard surgical options available for patients suffering from refractory urinary and/or fecal incontinence. This was a preliminary cost description, and hence therapy failure, future emergency visits, additional surgical and pharmacological treatments, and treatments for other disorders that might stem from incontinence (mental health), and the economic impacts of therapy failure, such as loss of work time were outside the scope of this study. However, the preliminary cost description presented here, together with the QOL findings reported in this paper, future studies and literature reports can be used for future cost-effectiveness analysis that accounts for health outcomes, community costs, and transition of health states. SNM therapy has already been shown to be highly effective, improves QOL of children and carries minimal complications (18, 23). In addition, it is possibly less burdensome for patients and caregivers than botulinum toxin injections, antegrade stomal irrigation, or retrograde transanal irrigation systems, although, direct comparisons in term of quality of life or costs between these various options are not available in the literature. On the other hand, it is important to consider and discuss potential limitations when presenting SNM to families as an alternative treatment, such as risk of lead displacement with growth, jumping, high contact sports and falls, and possible need for surgical revisions.

In cases where all other options have been exhausted, the potential use of orphan therapies, such as SNM, that may take a long time to receive regulatory approval should be considered. This was a single center study with a small convenience sample of children with refractory incontinence and as such might not be generalizable to other populations. The use of questionnaires instead of interviews limits our ability to provide in depth assessments of the impact of refractory non-neurogenic UI/FI on QoL. Despite these limitations, our study highlights the challenges associated with non-neurogenic refractory incontinence on children and their families, as well as the value of patient and caregiver feedback when searching for alternative treatment options. Our findings support further investigation of the use and effectiveness of therapies, such as SNM and TENS that have not yet received regulatory approval in Canada, as alternatives to surgery in children suffering from non-neurogenic UI/FI. These novel therapies may be key to reducing the burden of this complex condition on families and improving patient and caregiver QoL.

## Data Availability Statement

The raw data supporting the conclusions of this article will be made available by the authors, without undue reservation.

## Ethics Statement

The studies involving human participants were reviewed and approved by Hospital for Sick Children Research Ethics Board. Written informed consent to participate in this study was provided by the participants' legal guardian/next of kin.

## Author Contributions

JD, EM, and RV conceived and executed the project and wrote the preliminary draft. MP administered the survey to the participants. EM and MP performed the qualitative analysis. JD, EM, and MP wrote the paper. AA performed quantitative analysis. LR and FO'K performed cost-effectiveness analysis. DE, RF, AL, and MK contributed to the project conception and execution with MK supervising the project as well. All authors contributed to the article and approved the submitted version.

## Conflict of Interest

The authors declare that the research was conducted in the absence of any commercial or financial relationships that could be construed as a potential conflict of interest.
