# Supplementary material for: Assessment of Needs in Children Suffering From Refractory Non-neurogenic Urinary and Fecal Incontinence and Their Caregivers' Needs and Attitudes Toward Alternative Therapies (SNM, TENS)
Source: Front Pediatr. 2020 Sep 9;8:558. doi: 10.3389/fped.2020.00558 (PMC7509042; doi:10.3389/fped.2020.00558)
Supplement: Supplementary file 1 [file Data_Sheet_1.PDF]

Supplementary Information

Refractory non-neurogenic fecal and urinary incontinence in children: An assessment of needs

Joana Dos Santos MD MHSc[1,6*], Edyta Marcon PhD[2,6*], Martha Pokarowski[1*], Reza Vali MD MHSc[3,6], Lucshman Raveendran[1,4], Afsaneh Amirabadi PhD, MCCPM[3], Dean Elterman MD MSc[5], Richard Foty PhD[6], Armando Lorenzo MD MSc[1], Martin Koyle MD MSc[1,4]

Appendix A. Interview questions for patients.

1. Tell me what it is like to have wetting and/or poop accidents.
2. How do you manage?
3. How does it impact your life?

Appendix B. Interview questions for caregiver.

1. Tell me what it is like to have a child with bladder and/or bowel issues?
2. How do you manage?
3. How does it impact your life?
4. Have there been any financial implications?
5. What do you feel is the biggest burden on your child living with this condition?
6. How many therapies has your child tried to help treat bladder and/or bowel issues?
7. What are your expectations with treatment?
8. What has been your experience with the treatment and/or management of bladder and/or bowel issues?
9. What were the most significant side effects of the treatments?
10. What, in your opinion, would be the benefit (if any) of having a new treatment for bladder and bowel issues?
11. How open will you be to try TENS or SNS therapy?
12. Is there anything else you would like to add?
